# The Road to Quantitative Lipid Biochemistry in Living Cells

**DOI:** 10.1021/acs.accounts.2c00804

**Published:** 2023-03-21

**Authors:** Juan M. Iglesias-Artola, André Nadler

**Affiliations:** Max Planck Institute of Molecular Cell Biology and Genetics, Pfotenhauerstrasse 108, Dresden 01307, Germany

## Abstract

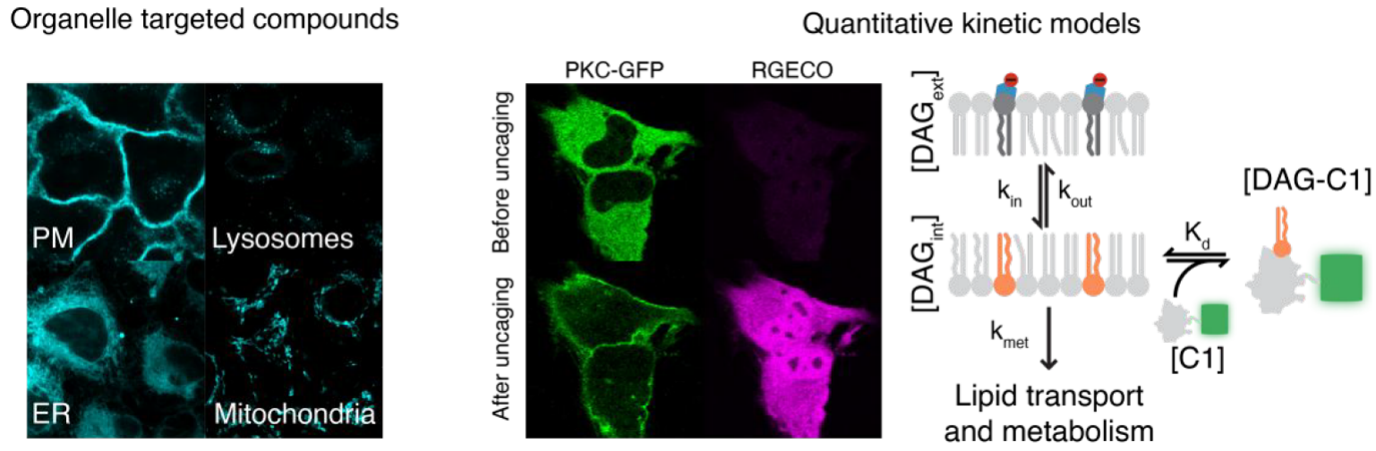

Traditional cell biological techniques are not readily suitable for studying lipid signaling events because genetic perturbations are much slower than the interconversion of lipids in complex metabolic networks. For this reason, novel chemical biological approaches have been developed. One approach is to chemically modify a lipid with a so-called “caging group” that renders it inactive, but this cage can be removed photochemically inside cells to release the bioactive molecule. These caged compounds offer unique advantages for studying the kinetics of cellular biochemistry and have been extensively used in the past. However, a limitation of conventional caged compounds is their ability to diffuse freely inside the cell, which does not permit localized activation below optical precision. This poses a challenge for studying lipid signaling as lipid function inside cells is tightly linked to their parent membrane. An ideal lipid probe should, therefore, be restricted to a single organelle membrane or preferentially to a single leaflet. We first demonstrated the plasma-membrane-specific photorelease of fatty acids by employing sulfonated caging groups. Using these caged fatty acid probes we demonstrated that lipid localization determines signaling outcome. Generalizing this approach, we designed a so-called “click cage” that can be coupled to lipids and offers the possibility to attach organelle targeting groups via click chemistry. Using this strategy, we have synthesized plasma membrane, lysosomal, mitochondria, and endoplasmic-reticulum-targeted lipids that can be used to dissect organelle-specific signaling events. To reduce the synthetic effort associated with generating caged compounds, we designed a coumarin triflate reagent that allows the direct functionalization of phosphate- or carboxylate-containing compounds. With this novel reagent, we synthesized a small library of photocaged G-protein-coupled receptor (GPCR) ligands to study the underlying lipid signaling dynamics. Most recently, we have focused on quantifying the kinetics of lipid signaling for different diacylglycerol (DAG) species using plasma-membrane-targeted caged DAGs. Using this approach, we quantitatively measured lipid–protein affinities and lipid transbilayer dynamics in living cells. After analyzing DAGs with different acyl chain length and saturation degree, we discovered that affinities can vary by up to an order of magnitude. This finding clearly shows that cells are able to distinguish between individual DAG species, thereby demonstrating that lipid diversity matters in cellular signal processing. Although the recent advances have yielded valuable tools to study lipid signaling, challenges remain on specifically targeting the different leaflets of organelle membranes. Furthermore, it is necessary to simplify the experimental approaches required for parametrizing and corroborating quantitative kinetic models of lipid signaling. In the future, we envision that the application of leaflet-specific caged lipids to model membrane systems will be of crucial importance for understanding lipid asymmetry.

## Key References

- NadlerA.; YushchenkoD. A.; MüllerR.; SteinF.; FengS.; MulleC.; CartaM.; SchultzC.Exclusive Photorelease of Signaling Lipids at the Plasma Membrane. Nat. Commun.2015, 6 ( (1), ), 100562668673610.1038/ncomms10056PMC4703838.^1^ Development of plasma membrane specific caging groups for fatty acids. Shows that fatty acid localization and function are linked. Release of arachidonic acid in the plasma membrane triggers Ca^2+^ oscillations in MIN6 cells, release in internal membranes results in downregulation.
- WagnerN.; StephanM.; HöglingerD.; NadlerA.A Click Cage: Organelle-Specific Uncaging of Lipid Messengers. Angew. Chem. Int. Ed.2018, 57 ( (40), ), 13339–1334310.1002/anie.201807497PMC617515930048020.^2^ Streamlined the synthesis of organelle-specific caging groups by introducing a butynyl unit in place of an ethyl group at the tertiary aromatic amine of the diethylaminocoumarin caging group.
- WagnerN.; SchuhmacherM.; LohmannA.; NadlerA.A Coumarin Triflate Reagent Enables One-Step Synthesis of Photo-Caged Lipid Metabolites for Studying Cell Signaling. Chem. Eur. J.2019, 25 ( (68), ), 15483–154873146118410.1002/chem.201903909PMC6916161.^3^ Show the in situ synthesis of diethylaminocoumarin-triflate to directly functionalize native lipids with a photocaging group at the phosphate or carboxylate moieties.
- SchuhmacherM.; GrasskampA. T.; BarahtjanP.; WagnerN.; LombardotB.; SchuhmacherJ. S.; SalaP.; LohmannA.; HenryI.; ShevchenkoA.; CoskunÜ.; WalterA. M.; NadlerA.Live-Cell Lipid Biochemistry Reveals a Role of Diacylglycerol Side-Chain Composition for Cellular Lipid Dynamics and Protein Affinities. Proc. Natl. Acad. Sci.2020, 117 ( (14), ), 7729–77383221358410.1073/pnas.1912684117PMC7149225.^4^ By making use of different caged diacylglycerol species (DAGs), the authors show that the structural differences in DAGs are relevant for PKC signal specification. Importantly, the authors calculated that protein-affinities for two native DAGs differ by an order of magnitude.

## Introduction

Lipids perform three main functions in cells. They constitute limiting membranes of individual organelles and the entire cell, they are the primary cellular means for long-term energy storage, and they are key components in signaling pathways as receptor ligands and second messengers.^5^ To perform these different functions, numerous kinds of lipids are synthesized by the cell. They can be subdivided in categories, including fatty acids, glycerolipids, phosphoglycerolipids, sphingolipids, and sterols.^6^ Each lipid category is composed of several lipid classes that differ in their specific hydrophilic headgroup. Individual lipid classes in turn feature numerous lipid species that differ in acyl chain length and saturation degree. Taken together, this provides mammalian cells with a chemical space in which several thousands of different lipid species can be realized.^7,8^ Mass spectrometric analysis has confirmed that mammalian cells do, in fact, produce many hundreds, if not thousands, of the theoretically possible lipid species.^9^ The realization of the chemical complexity of the cellular lipidome has given rise to the term lipid diversity and in turn triggered questions about the biological roles of these molecules.

The cell biology of lipids is best understood at the level of lipid classes. Various phosphatidylinositides contribute to defining organelle identities and play a role in signaling cascades.^10,11^ For example, phosphatidylcholines are the main membrane-forming lipids,^12^ and cardiolipins ensure the functioning of the unique inner mitochondrial membrane.^13^ Furthermore, we roughly know how lipid classes are distributed between organelles^5^ and even individual membrane leaflets.^14^ However, lipidomic analyses of diseased tissue,^15^ plasma samples,^16,17^ and also genetically modified cells^18^ often reveal significant changes on the level of individual lipid species rather than entire lipid classes. Such findings strongly suggest the existence of cellular regulatory mechanisms that control the level of individual, molecularly distinct lipid species. In turn, biological function would be associated with lipid species, rather than with entire lipid classes. However, mechanistic analysis of the role of individual lipid species in cell biology is still severely constrained by the available methodological repertoire that can be brought to bear on the problem. Unlike proteins, lipids cannot be faithfully visualized by attaching fluorescent tags—the chemical changes introduced by such modifications far outweigh the chemical differences between individual lipid species from the same class. Furthermore, genetic perturbations are a very indirect way to modulate lipid levels, as the consequences of removing components of the lipid handling machinery are difficult to predict.

Because traditional cell biological techniques are less suited to study the functions of lipids, numerous chemical biology approaches have been developed in order to expand the methodological repertoire. These tools include chemical dimerizer approaches,^19−21^ photocaged^22^ and photoswitchable lipids^23−28^ for modulating lipid levels, and a number of applications of bioorthogonal chemistry for the visualization of lipid metabolism and localization and the study of the lipid–protein interactome.^29−34^ Within this broader push by the chemical biology community toward the development of more accurate methods for studying lipids, our laboratory has focused on developing photocaged lipid probes that allow one to perturb the levels of individual lipids in an organelle in a specific and quantitative manner. The present Account details our efforts over the last years in this space.

## Main Text

Caged compounds are bioactive molecules that are rendered inactive by attaching a photoremovable protecting group that inhibits their biological activity.^35−38^ They are applied to biological samples in their inactive form and are activated by photorelease of the bioactive molecule through photolysis. Photolysis is initiated during the course of an experiment, e.g., a fluorescence microscopy time lapse or an electrophysiological recording. Because the photolysis reaction is typically orders of magnitude faster than the investigated biological process, and because the cellular response is triggered only by the unmodified bioactive compound after photolysis, caged compounds offer unique advantages for studying the kinetics of cellular biochemistry and have been extensively used in that role for decades.

**Figure 1:**
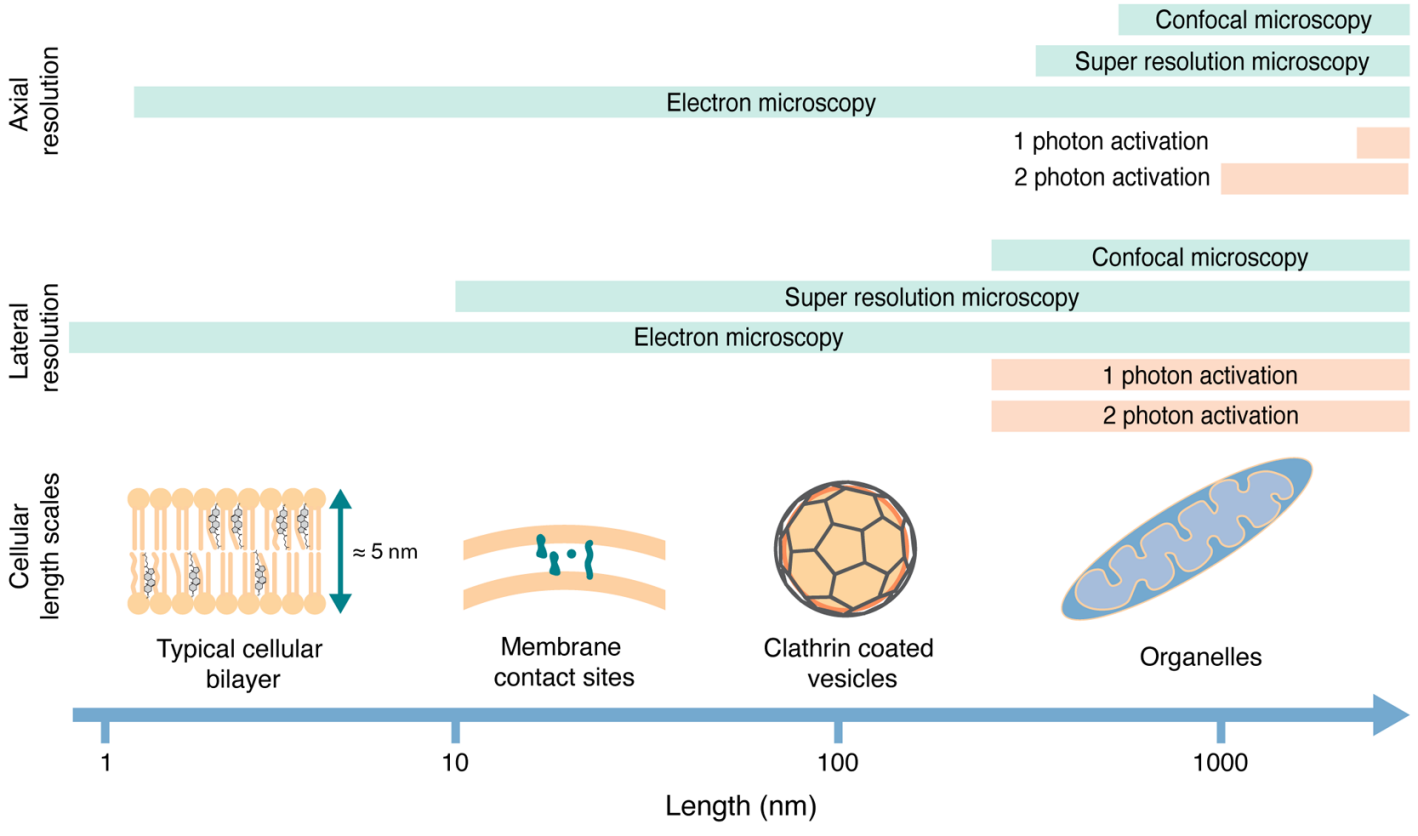
Intracellular organization spans several length scales. Visualization techniques give different levels of resolution (green bars). Optical perturbations (orange bars) have a limited resolution that does not permit exclusive perturbation of individual organelles.

Most caged compounds are soluble small molecules, in particular, neurotransmitters.^39−42^ They are either applied extracellularly or converted into bioactivatable prodrugs through, e.g., functionalization with acetoxymethyl (AM) groups to convert them to AM esters that are cell permeable and allow delivery into the cytoplasm prior to photoactivation. In both cases, they are able to rapidly diffuse through space (as their parent molecules), and the question of localized activation on submicron scales is largely irrelevant since rapid, largely unhindered diffusion in three dimensions is an inherent element of their mode of action. Thus, the precision that can be achieved by optical means (300 nm in *x*–*y*, 1500 nm in *z* for 1-photon activation, in the low hundreds of nanometers for 2-photon activation) is typically sufficient for initiating local perturbations in cell biological experiments (Figure 1).

This is not strictly true for the application of caged lipids. Lipids act mostly in cellular membranes, and the investigation of their biological functions necessitates taking the cellular architecture into account. The membranes of cellular organelles feature distinct lipid compositions that furthermore differ in the degree of asymmetric lipid distribution between the membrane leaflets.^14^ For this reason, any given lipid can have divergent roles in different organelle membranes. A good example is diacylglycerol (DAG), a lipid that acts as a second messenger during cell signaling at the plasma membrane.^43,44^ DAG can also serve as the key intermediate for glycerophospholipid biosynthesis in the membrane of the endoplasmic reticulum^45^ and is a catabolic intermediate during the mobilization of energy stored in the form of triacylglycerides in lipid droplets.^45,46^

To avoid affecting multiple unrelated processes in the same experiment, lipid photoactivation should preferentially be restricted to a single organelle membrane, ideally to a single leaflet. Distinct cellular membranes come as close as 15–20 nm in membrane contact sites, whereas individual membrane leaflets have an average width of 2.5 nm. These dimensions suggest that photoactivation in specific cellular membranes is not possible using current optical technology if the caged lipid is distributed through all cellular membranes. The problem of localized lipid uncaging thus necessitates a chemical solution—caged lipids need to be prelocalized in the membrane/compartment of interest prior to photoactivation. Since, in this case, photoactivation can only take place where the caged compound resides, off-target light will not have any effects if careful controls for phototoxicity are in place.

The development of organelle-specific caged lipids was initiated by one of us (A.N.) together with colleagues in the Schultz laboratory in 2015^1^ and has been driven forward by our laboratory and others (in particular, the laboratories of Höglinger,^47^ Riezman,^48,49^ Yushenko^50,51^ and Frank^52^) since then. Here, we focus mainly on our contributions to the field, retrace the development and applications of prelocalized caged lipids during the last years, and offer a perspective for future directions.

The decision to develop organelle-specific approaches for modulating the levels of molecularly distinct lipid species with light was motivated by our finding that individual diacylglycerol species have different abilities to trigger localized signaling responses in uncaging experiments^53^ and the realization that simple fatty acids, such as arachidonic acid, fulfill highly specialized functions in small signaling compartments.^54^

**Figure 2:**
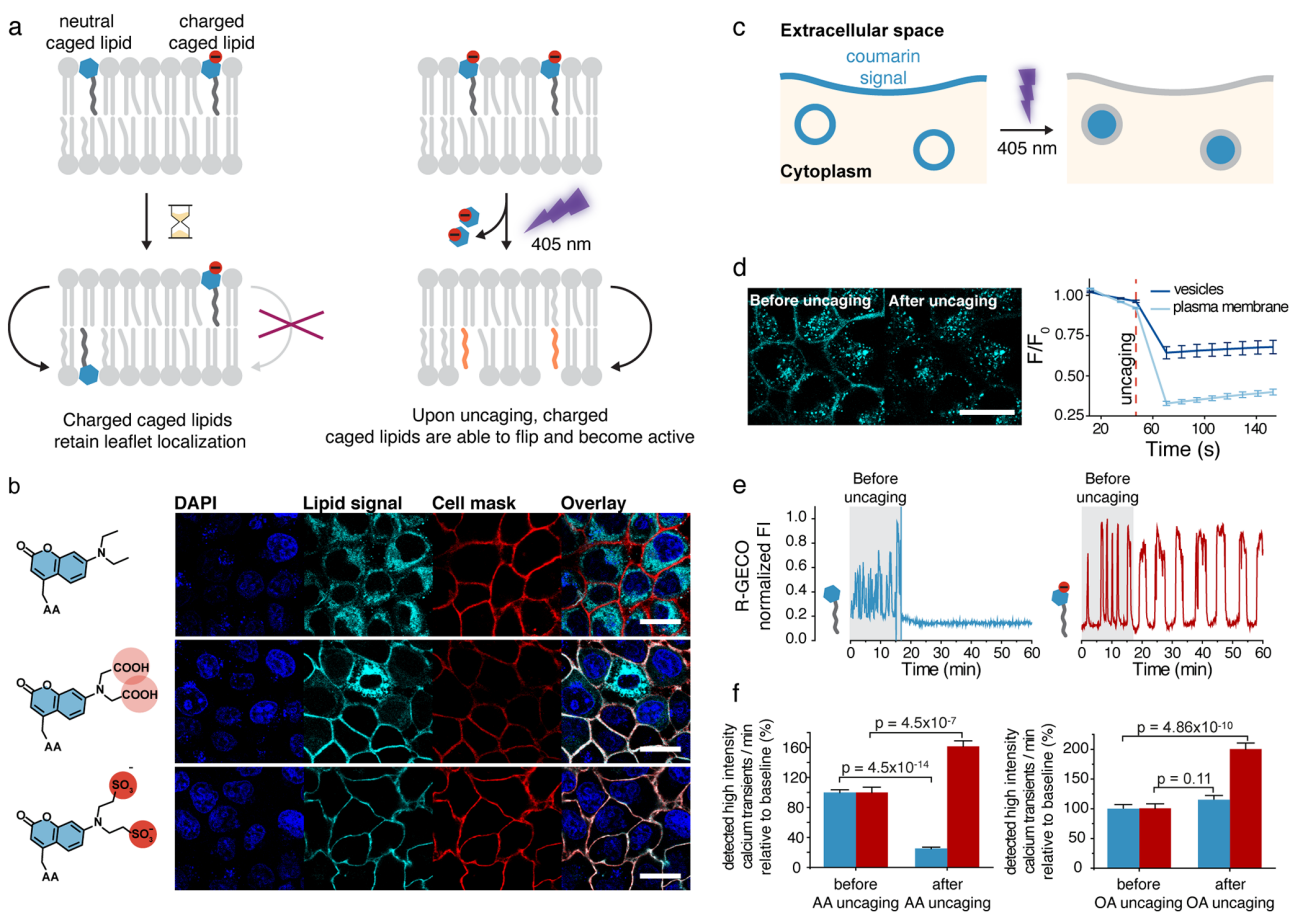
Intracellular localization of the lipid photorelease is relevant for the signaling outcome. (a) Concept of leaflet-specific caged lipids. Charged caged lipids are unable to flip to the inner leaflet of the plasma membrane. Upon photolysis, the caging group is released, and the lipid becomes active and is able to flip to the intracellular leaflet. (b) Modification of the diethylaminocoumarin (DEAC) group allows for specific localization of the probe to the plasma membrane, with the sulfonated compound showing the highest plasma membrane specificity. (c) Concept of coumarin distribution after photolysis. Given that the sulfonated caged lipid is exclusively localized in the outer leaflet of the plasma membrane, release of the caging group results in a decrease of signal as the coumarin signal diffuses in the medium. After endocytosis, the lipid is present in the inner leaflet of the intracellular vesicles; therefore, photorelease of the coumarin group results in a release of the DEAC group into the lumen, and the decrease in signal results from bleaching. (d) Exemplary images of uncaging experiments and image quantification. The left panel shows the ratio of fluorescence intensity over initial fluorescence intensity as a function of time depending on the localization of the lipid probe. (e) Calcium transients in MIN6 cells measured by an R-GECO signal. The left panel shows the cellular response upon uncaging arachidonic acid without a specific cellular localization. The right panel shows the cellular response upon uncaging arachidonic acid exclusively at the plasma membrane. (f), Distribution of high-intensity calcium events for arachidonic acid (left panel) and oleic acid (right panel). Blue bars show the result of uncaging the nonspecific lipid probe; red bars show the result of uncaging the plasma-membrane-specific lipid probes. Scale bars: 20 μm. Adapted with permission from ref (1). Copyright 2015 The Authors. Published by Wiley under a Creative Commons Attribution-NonCommercial 4.0 International (CC BY-NC 4.0) license.

We first set out to develop strategies that would enable us to deliver caged lipid probes specifically to the plasma membrane. We focused on two fatty acids, arachidonic acid and oleic acid, as first proof-of-principle examples to demonstrate the feasibility of this approach. Both molecules serve as first and second messengers in cellular signaling besides their role as crucial intermediates in lipid biogenesis and energy metabolism. Since any chemical probe has to move across the plasma membrane to reach the cytoplasm, we reasoned that modifications of the caging group could block transbilayer movement and would allow us to generate probes that exclusively localize to the outer leaflet of the plasma membrane prior to uncaging–provided that endocytosis is sufficiently slow. Specifically, the introduction of negative charges on the utilized caging group should prevent caged lipid probes from crossing the plasma membrane, as the hydrophobic side chains would incorporate into the outer leaflet, but flipping would be thermodynamically highly unfavorable (Figure 2a). To achieve this, we modified the common diethylaminocoumarin (DEAC) caging group with either carboxylate or sulfonate substituents and used arachidonic acid derivatives equipped with either the unmodified or the modified photocaging group to assess cellular localization (Figure 2b). Using the intrinsic fluorescence of the coumarin caging groups as a readout, we found that the probe bearing the original DEAC group was completely internalized, whereas the carboxyl-modified probe exhibited a mixed localization between internal membranes and the plasma membrane and significant cell-to-cell heterogeneity, thereby making it unsuitable for caged lipid prelocalization. The sulfonated coumarin derivative exhibited the desired, plasma-membrane-specific localization (Figure 2b). The differences in plasma membrane retention between the sulfonated and carboxylated versions of the probes can likely be explained by the lower p*K*_a_ value of the sulfonated compound—a small but sufficient portion of the carboxylated caging group is protonated at any given time and can, thus, passively cross the plasma membrane with a nonzero rate, which is not the case for the sulfonated probe.

Using these probes, we found that plasma membrane localization was largely maintained in Hela and MIN6 cells for 30–60 min after the initial loading procedure, after which significant probe internalization by endocytosis became observable (Figure 2b). The mixed endosomal and plasma membrane localization proved to be useful for quantifying uncaging photoreactions in living cells by monitoring the intrinsic fluorescence of the coumarin caging group. Upon photocleavage at the plasma membrane, the coumarin is released to the extracellular space and subsequently quickly diluted; a completed photoreaction is, thus, indicated by a loss of fluorescence. In contrast, release from the inner endosomal membrane releases the coumarin into the endosomal lumen, where it remains trapped (Figure 2c,d). Successful photoreactions, thus, do not elicit any change in fluorescence intensity in time-lapse imaging experiments because endosomal lumen and membrane are not resolved in confocal imaging (Figure 2c,d). A loss of fluorescence in the endosomal compartment is, thus, only indicative of bleaching. These measurements can be used to optimize the light doses required for the efficiency and completeness of the photoreactions and help prevent photostimulation artifacts by avoiding overstimulation.

Plasma-membrane-specific caged lipids can be used in combination with their nonspecific counterparts to investigate whether the intracellular localization of the lipid messenger photorelease is relevant for the signaling outcome. We chose to address this question in two biological systems: calcium signaling in insulin-secreting beta cells (using the MIN6 model cell line) and the modulation of synaptic transmission in mossy fiber to CA3 synapses, which we analyzed in mouse brain slices. Beta cells exhibit spontaneous calcium transients if glucose is present in the medium, which often display oscillatory patterns. Upon uncaging of either arachidonic acid or oleic acid on the plasma membrane, calcium transients were both more pronounced and more frequent (Figure 2e,f). In contrast, arachidonic acid uncaging on internal membranes led to a complete inhibition of calcium signaling and, by extension, insulin release, whereas oleic acid uncaging on internal membranes had no observable effects (Figure 2e,f). The stimulatory effect of plasma membrane photorelease of both long-chain fatty acids is likely due to activation of the free fatty acid receptor GPR40, as demonstrated by pharmacological inhibition, and in line with the broad agonist spectrum of this G-protein-coupled receptor (GPCR). The arachidonic-acid-specific inhibition of calcium oscillation after photorelease on internal membranes appears to occur via a not yet identified specific interactor and is independent from endocannabinoid signaling, as inhibition of endocannabinoid receptors did not affect the acute inhibition of calcium transients.

In a complementary set of experiments, we used arachidonic acid uncaging at the plasma membrane and internal membranes in mossy fiber to CA3 synapses and performed whole-cell voltage clamp recordings of excitatory postsynaptic currents. We found that photorelease of arachidonic acid at the plasma membrane resulted in sustained potentiation of synaptic transmission, whereas photorelease on internal membranes had a significantly smaller effect. This finding is in line with the direct action of arachidonic acid on presynaptic Kv channels.

Taken together, this first study on organelle-specific lipid uncaging demonstrated that plasma-membrane-targeted probes can be obtained by decorating the caging group with sulfonates, which prevents lipid flipping. We showed that these probes can be used to monitor uncaging photoreactions using fluorescence microscopy because they remain in the exoplasmic leaflet of all cellular membranes. These first biological applications clearly demonstrated that the site of lipid photorelease is of great importance for ultimate signaling outcome. We further demonstrated that the photorelease of arachidonic acid can be either stimulatory (on the plasma membrane) or inhibitory (on internal membranes) for MIN6 calcium oscillations. Together, these findings highlighted the need for developing finely tailored lipid probes designed to perturb and study individual cellular compartments.

Our next aim was to increase the flexibility of organelle-targeted lipids. Besides our work on plasma-membrane targeted lipids, the Riezmann laboratory reported probes that could be directed to the mitochondria.^48^ However, both probe types and other theoretically accessible probes require dedicated synthetic routes, thereby limiting their application potential. This speaks to a general problem in chemical biology. As probes become ever more sophisticated to account for the requirements of complex cell biological experiments, the synthetic effort to generate probes increases. This leads to a point where probe syntheses are only carried out for a designated project and not repeated for follow-up studies. Thus, streamlined strategies for generating highly specialized probes are warranted.

**Figure 3:**
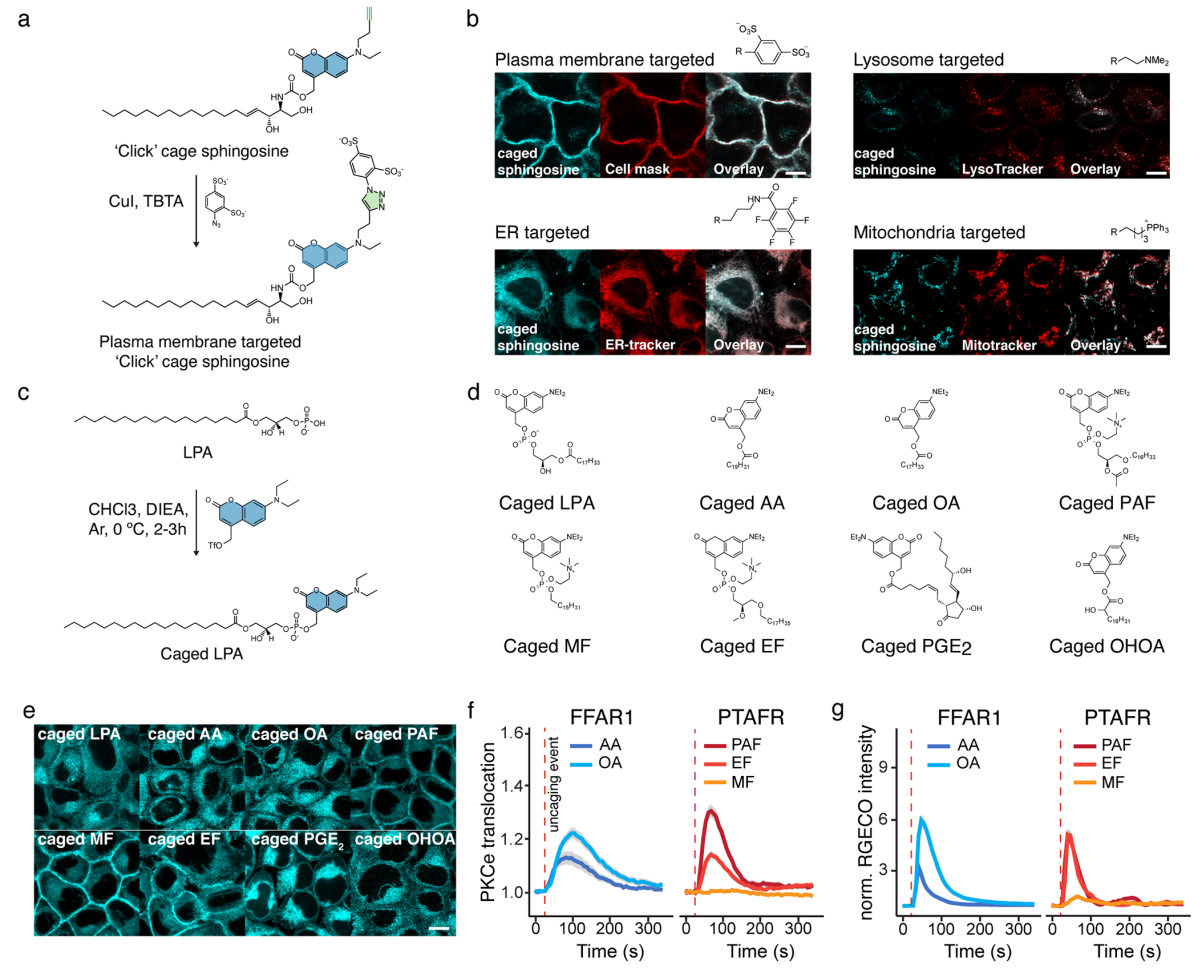
New synthetic strategies to produce complex caged lipids. (a) The use of a “click cage” facilitates the addition of organelle-targeting groups to caged lipids. (b) Microscopy images of caged sphingosine with different organelle-targeting groups. Adapted with permission from ref (2). Copyright 2018 The Authors. Published by Wiley under a Creative Commons Attribution-NonCommercial 4.0 International (CC BY-NC 4.0) license. (c) A DEAC-triflate can be use to functionalize native lipids with a photocaging group at the phosphate or carboxylate moieties. (d) Library of caged compounds generated using DEAC-triflate. (e) Microscopy images of the different caged lipids in cells. Images were acquired using the coumarin fluorescence. (f) Protein kinase C epsilon (PKCε) recruitment to the plasma membrane upon lipid uncaging in HeLa cells expressing FFAR1 (left panel) or PTAFR (right panel) over time. (g) Ca^2+^ signal measured by RGECO fluorescence over time in HeLa cells expressing FFAR1 (left panel) or PTAFR (right panel). Scale bars: 10 μm. use Adapted with permission from ref (3). Copyright 2019 The Authors. Published by Wiley under a Creative Commons Attribution-NonCommercial 4.0 International (CC BY-NC 4.0) license.

Motivated by these considerations, we designed a so-called “click cage” photocaging group to allow modular assembly of caged lipid probes targeted to different intracellular compartments.^2^ On the basis of the DEAC scaffold, the “click cage” is equipped with a butynyl unit in the place of an ethyl group at the tertiary aromatic amine (Figure 3a). The “click cage” group can be coupled to biomolecules using protocols previously established for the DEAC group, as demonstrated by us for arachidonic acid and sphingosine (Figure 3a). The alkyne handle allows the attachment of various organelle-directing groups as the last step of probe synthesis by copper-mediated [3 + 2] cycloaddition reactions, which allows the generation of an entire set of organelle-directed caged lipids for various cellular compartments with minimal additional effort. Specifically, we used the triphenylphosphonium cation to target probes to the mitochondria, a bisulfonated phenyl group to generate plasma-membrane-directed probes, a tertiary amine to synthesize caged lipids that reside in the exoplasmic leaflet of the lysosomal membrane, and pentafluorophenyl groups to target caged lipids to the endoplasmic reticulum (ER) membrane (Figure 3b). Correct intracellular localization of the probes was assessed by colocalization with known organelle markers. Our probes exhibited a high Pearson correlation coefficient with the organelle markers of at least 0.72. Interestingly, we found that caged lipids targeted to the mitochondria, the plasma membrane, and the lysosomes exhibited good cellular uptake rates, whereas the perfluorinated ER-targeted probes were barely taken up. This observation points to a potential problem with using perfluorinated groups to direct lipids and other small molecules to the ER because it may prove difficult to deliver probes in sufficient amounts to elicit cellular responses.

To demonstrate the biological applicability of the generated probes, we conducted calcium imaging experiments in HeLa cells loaded with Fluo-4. We found that calcium transients depended both on the lipid messenger structure and the intracellular site of uncaging. This was best demonstrated by the uncaging of sphingosine and arachidonic acid at the plasma membrane: arachidonic acid triggered robust, likely GPR40-mediated responses, whereas sphingosine uncaging had no effect. In contrast with this finding, sphingosine uncaging in the lysosomes triggered much stronger calcium transients compared with the respective arachidonic acid experiments. In summary, we demonstrated modular synthesis of entire sets of caged lipids for various cellular compartments and their application for calcium imaging in proof-of-principle experiments.

In order to further streamline the generation of caged lipid probes, we then set out to develop reaction methods that would allow us to directly couple a photocaging group to native lipids in a single-step reaction sequence. This poses a difficult synthetic problem because lipid headgroups often feature multiple functional groups with relatively low nucleophilicity, primarily hydroxyl groups and carboxylate and phosphate moieties. Furthermore, because photorelease kinetics of caged phosphate or carboxylates are typically much more favorable than similar reactions involving hydroxyl groups, a reaction for functionalizing the former two functional groups in the presence of free hydroxyl groups would be most useful. Such transformations have been carried out in a limited number of cases using diazo reagents, but low reactivity has limited the applications. We, thus, postulated that benzylic triflate reagents (Figure 3c) should be sufficiently reactive to functionalize even difficult, zwitterionic starting reactants. Reagent stability was a concern since only a single functionalization reaction involving a benzylic triflate reagent had been reported before.^55^ Nevertheless, upon generation of DEAC-triflate in situ and direct use of the reaction mixture for the functionalization reaction, we found that native lipids could indeed be selectively equipped with a photocaging group at the phosphate or carboxylate moieties.^3^ We demonstrated the utility of this transformation for a number of potent G-protein-coupled receptor (GPCR) ligands, including lysophosphatidic acid, platelet activating factor, and prostaglandin E2. After synthesizing an entire set of photocaged GPCR ligands (Figure 3d,e), we proceeded to compare signaling responses to various receptor/ligand pairs. We could clearly demonstrate that GPR40 activation by arachidonic acid induced more intense and longer-lasting calcium and protein kinase C epsilon (PKCε) responses than oleic acid photoactivation (Figure 3 f,g). A comparison of the effects of platelet-activating factor uncaging in platelet-activating factor receptor (PTAFR)-expressing cells and its synthetic analogues revealed that the platelet-activating factor (PAF) analogue edelfosin elicited similar calcium transients but greatly diminished PKCε activation, whereas the structurally more different analogue miltefosin did not trigger any signaling responses (Figure 3 f,g). Taken together, we demonstrated the power of a new reagent to facilitate the rapid generation of small libraries of photocaged GPCR ligands that can be used for studying the underlying lipid signaling dynamics in a comparative approach.

Our work on lipid signaling up to this point can be characterized as semiquantitative, which is still the standard for the field. This is because the absolute density of lipids in living cells is notoriously difficult to quantify, especially on subcellular scales. However, for describing lipid signaling in quantitative terms, it is necessary to either measure or infer lipid densities in individual membrane leaflets, as this is how they are produced and regulated in the cell. We reasoned that diacylglycerol signaling would be a suitable proof-of-principle example for developing a quantitative methodology to analyze the role of lipids in cellular signaling events.

Signaling diacylglycerols (DAGs) are generated and signal at the plasma membrane after cell surface receptor activation. They recruit effector proteins to the plasma membrane, mostly through DAG-C1 domain interactions. In turn, these effector proteins subsequently propagate the cellular signal to nodes further downstream in the signaling network. In a secondary mode of action, DAGs can also function as an agonist for plasma-membrane-localized calcium channels, including the transient receptor potential (TRP) channels TRPC3 and TRPC6.

**Figure 4:**
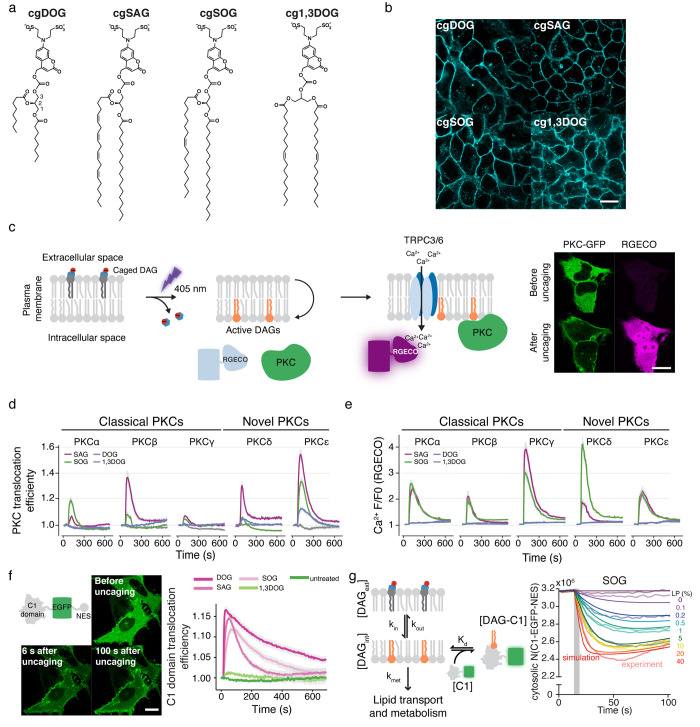
Cells are able to distinguish small chemical differences between DAG species. (a) Chemical structures of the different caged DAGs used. (b)Representative fluorescent images of the caged DAGs loaded into the plasma membrane of HeLa cells. (c) Experimental concept. Upon uncaging, caged lipids flip to the inner leaflet of the plasma membrane where they activate TRPC3/6 channels that allow Ca^2+^ to enter the cell and recruit PKCs to the plasma membrane. The increase of Ca^2+^ in the cytoplasm increases the fluorescence of the calcium sensor RGECO. The right panel shows representative images of the cells before and after uncaging. (d) Quantification of the translocation of the different PKC isoforms to the plasma membrane over time by the different DAG species (line colors). (e) Mean normalized fluorescence intensity of the RGECO calcium sensor over time in cells overexpressing the different PKC isoforms with the different DAG species (line colors). (f) Left panel, timelapse of the recruitment of the C1-EGFP-NES construct to the plasma membrane after DAG uncaging. Right panel, C1-EGFP-NES translocation efficiency to the plasma membrane over time for the different DAG species (line colors). (g) Left panel, kinetic model of C1 recruitment to the plasma membrane by DAGs. Right panel, number of free C1-EGFP-NES proteins over time for SOG upon uncaging using different laser powers (LP). Faint lines represent the measured amount of free C1-EGFP-NES, darker lines represent the prediction by the model with the estimated fit parameters. Scale bars: 20 μm. Adapted with permission from ref (4). Copyright 2020 The Author(s). Published by PNAS. This open access article is distributed under Creative Commons Attribution-NonCommercial-NoDerivatives License 4.0 (CC BY-NC-ND).

We first generated a set of three signaling active, caged DAGs,^4^ including the arachidonate-containing variant caged 1-stearoyl-2-arachidonoyl-*sn*-glycerol (cgSAG), which is considered to be the archetypical signaling DAG; the oleic acid containing caged species 1-stearoyl-2-oleoyl-*sn*-glycerol (cgSOG), which corresponds to one of the most prevalent DAG species in mammalian cells; and the short chain, soluble caged DAG analogue 1,2-dioctanoyl-*sn*-glycerol (cgDOG), which does not correspond to a native DAG species but has nevertheless been heavily used in cell biological experiments (Figure 4a,b). To control for general phototoxicity and other undesired effects of the uncaging photoreaction, we also synthesized the inactive caged regioisomer 1,3-oleoyl-*sn*-glycerol (cg1,3DOG), which faithfully replicates the photoreaction but does not induce signaling responses at the plasma membrane (Figure 4a,b).

Using the same approach as in our 2015 paper,^1^ we next established that similar plasma membrane incorporation and photorelease efficiencies could be reached for all compounds by monitoring coumarin fluorescence intensity during uncaging experiments. While this alone does not allow for statements to be made about the absolute number of photoreleased DAG molecules on a given fraction of a plasma membrane surface, it did allow us to test whether the photorelease of the similar amounts of structurally different DAGs leads to varying downstream signaling patterns. We reasoned that such a finding would provide enough justification for a drive toward establishing absolute quantification of lipid uncaging experiments, which we expected to be a major undertaking of uncertain outcome. We, thus, performed DAG uncaging experiments and monitored the plasma membrane recruitment of a set of classical (α, β, γ) and novel (δ, ε) protein kinase C isoforms, important signaling effector proteins that all contain DAG-binding C1 domains (Figure 4c). Simultaneously, we measured intracellular calcium levels to account for DAG-induced activation of TRP channels (Figure 4c). We found that the observed signaling transients were strongly affected by the chemical structure of the respective DAGs (Figure 4d). Specifically, novel (calcium-insensitive) PKC isoforms were recruited much more efficiently to the plasma membrane by the polyunsaturated SAG than they were recruited by both SOG and DOG (Figure 4d). Furthermore, the short-chain analogue soluble DOG did not elicit any calcium transients at all under these conditions, which indicated that it is a much less effective agonist for TRP channel activation than the native long-chain DAGs (Figure 4e). This was also reflected in the fact that classical PKCs, which require both calcium and DAG for plasma membrane recruitment and activation, did not translocate to the plasma membrane upon DOG uncaging. We furthermore observed interesting differences in the capacity of the native DAGs, SAG and SOG, to recruit classical PKC isoforms. SOG was significantly more efficient in recruiting PKCα than SAG, whereas the opposite was found to be true for PKCβ. Since the underlying calcium transients were virtually identical for both lipids, this suggested that DAG–protein interactions are responsible for the observed disparities in effector protein recruitment.

Having firmly established that structural differences between DAGs are relevant for signal specification, we set out to develop a methodology that would enable us to study the mechanistic reasons for the observed effects in quantitative kinetic experiments. We first constructed a minimal DAG binding protein (C1-EGFP-NES), a fusion protein composed of a C1 domain, EGFP, and a nuclear exclusion signal, which was included to prevent plasma membrane recruitment kinetics from being obscured by the slow release of a nuclear protein pool (Figure 4f). In order to determine the absolute number of protein molecules per cell, we purified the C1-EGFP-NES protein and generated a calibration curve to convert cytosolic fluorescence intensities into absolute concentrations. To estimate the absolute number of DAGs released at the plasma membrane, we first generated a calibration curve using giant unilamellar vesicles (GUVs) with a defined amount of caged DAGs and confirmed accurate incorporation by mass spectrometry. We then prepared giant plasma membrane unilamellar vesicles (GPMVs) from cells loaded with caged diacylglycerols and compared their fluorescence intensities with those of the previously prepared GUVs, which enabled us to calculate the caged DAG density at the plasma membrane of the loaded cells. The absolute number of caged DAGs incorporated into the plasma membrane per cell were determined by multiplying the obtained density with a measurement of the cell surface area. In combination with the measurement of the photorelease efficiency described above, this allowed us to estimate the absolute number of photoreleased DAG molecules per cell.

We next conducted DAG uncaging experiments in a light dose-dependent manner and acquired protein recruitment traces for a wide range of initial levels of photoreleased DAGs to, in effect, establish a quantitative, kinetic dose–response curve for DAG-driven protein recruitment to the plasma membrane. We used these data to conduct a parameter optimization for a minimal, kinetic model that describes DAG transbilayer movement, DAG–protein affinities, and DAG turnover. Despite the fact that the model contained only four free parameters, we found that it could explain the obtained data very well, and we were able to identify values for rate constants and affinities for all analyzed DAGs (Figure 4g). We found that the rates observed for lipid transbilayer movement differed significantly between species because the short-chain analogue DOG exhibited much faster dynamics than both long-chain derivatives. However, turnover of the long-chain derivatives was found to be much quicker. Most striking, however, was the finding that the lipid–protein affinities differed by an order of magnitude between the two native, long-chain DAGs. Taken together, these quantitative findings demonstrate that the cellular signaling machinery is able to distinguish small chemical differences between individual DAG species and, thus, lend credence to the argument that chemical differences between signaling lipid species are, indeed, functionally relevant for information encoding during cellular signal propagation.

## Summary and Outlook

In summary, our work over the last years has led to the generation of caged lipid probes that now can be used to modulate the levels of native lipid species in an organelle and, in some cases, in a leaflet-specific manner in living cells. By developing modular synthetic routes and new synthetic methodologies, we have made these tools readily accessible to explore the chemical space of the diverse cellular lipidome. In combination with quantitative microscopy approaches, the organelle-specific uncaging of lipids can be employed to perform quantitative lipid biochemistry in living cells. Such a capability is essential for studying the biological function of lipids on the species level, as shown in the example of signaling DAGs. Despite these advances, major hurdles remain. For now, only plasma membrane lipids can be modulated in a leaflet-specific manner, as demonstrated by us and the Frank laboratory for the outer leaflet.^52^ Leaflet-specific tools for the other organelles would, thus, be of great interest. Furthermore, while photoreactions can be quantified in principle in living cells, it requires significant experimental effort. Ideally, methods should be developed to infer the initial photoreaction yield from fitting kinetic models preferentially on a single cell level. Finally, we and all other laboratories in the field have had a strong focus on signaling lipids. While this reflects the fact that small absolute concentration changes will lead to strong cellular responses and minimize the probability of adverse effects of the induced photoreactions, it also constitutes a systematic bias with regard to the investigated part of the cellular lipidome. Thus, tools for studying the role of perceived “structural” lipid species should constitute a primary focus of future probe development efforts. The application of such probes should not be limited to cells but expanded to model membrane systems to complement applications of photoswitchable lipids. Leaflet-specific caged lipid probes, in particular, could prove invaluable for generating realistic model membrane systems for the study and ultimately understanding of lipid asymmetry.
